# Supplementary material for: The interactome of intact mitochondria by cross-linking mass spectrometry provides evidence for coexisting respiratory supercomplexes
Source: Mol Cell Proteomics. 2017 Dec 8;17(2):216–32. doi: 10.1074/mcp.RA117.000470 (PMC5795388; doi:10.1074/mcp.RA117.000470)
Supplement: Supplemental Data [file supp_RA117.000470_133922_0_supp_23978_fzffwf.zip › spectra_annotation/mito_spectra_annotation/147-1-3-1-16-1.pdf]

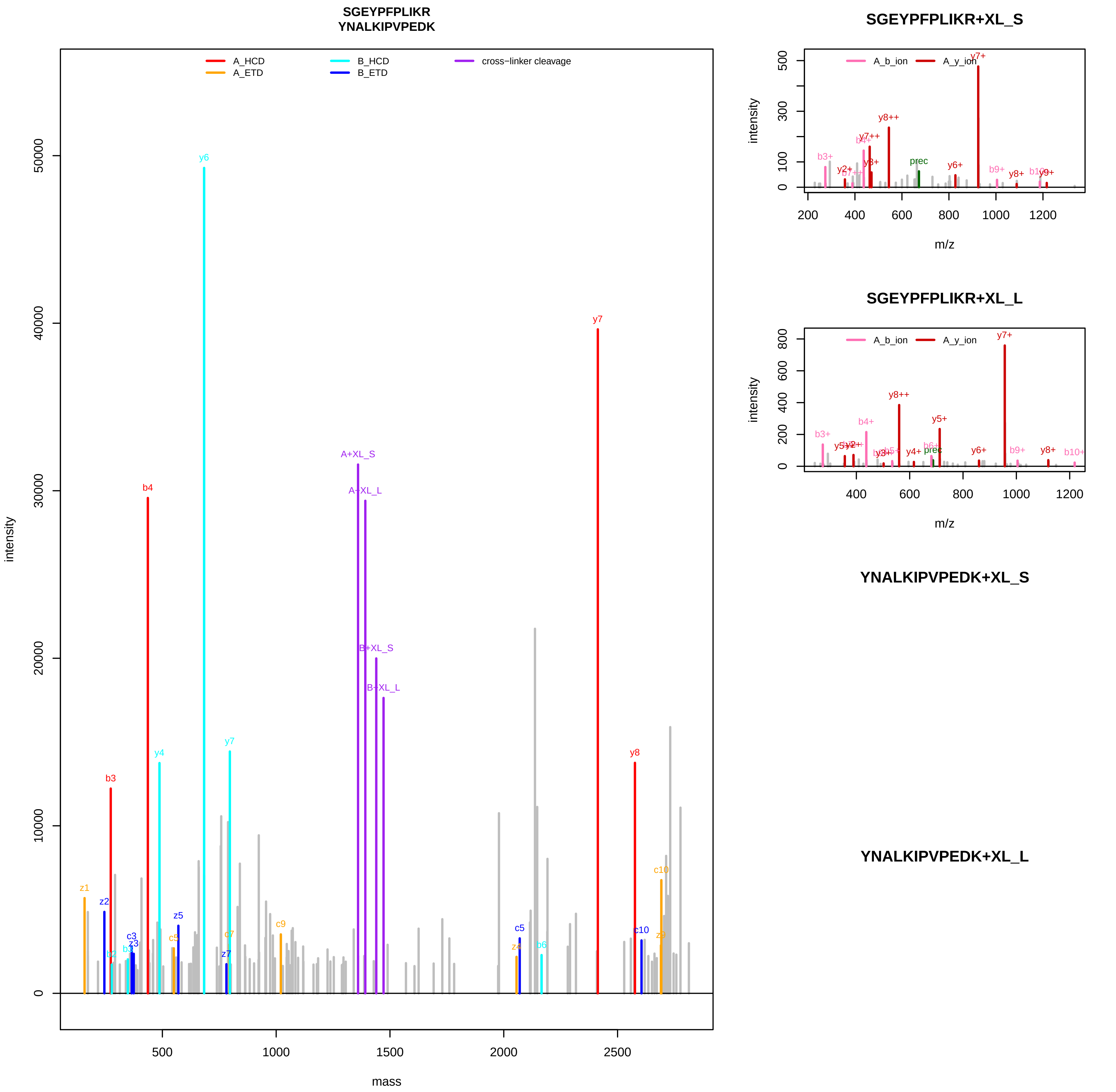
SGEYPFPLIKR
YNALKIPVPEDK
A_HCD
A_ETD
B_HCD
B_ETD
cross−linker cleavage
intensity
mass
y6
y7
b4
A+XL_S
A+XL_L
B+XL_S
B+XL_L
y4
y7
b3
y8
c10
z1
z2
z5
c3
z3
c5
c7
z7
c9
c5
z4
b6
c10
z9
SGEYPFPLIKR+XL_S
A_b_ion
A_y_ion
y7+
y8++
y7++
b4+
y3+
y2+
b3+
prec
y6+
b9+
y8+
y9+
m/z
SGEYPFPLIKR+XL_L
y8++
b4+
y5+
b3+
y4+
b6+
y6+
b9+
y8+
b10+
YNALKIPVPEDK+XL_S
YNALKIPVPEDK+XL_L
